# New Detection Systems of Bacteria Using Highly Selective Media Designed by SMART: Selective Medium-Design Algorithm Restricted by Two Constraints

**DOI:** 10.1371/journal.pone.0016512

**Published:** 2011-01-27

**Authors:** Takeshi Kawanishi, Takuya Shiraishi, Yukari Okano, Kyoko Sugawara, Masayoshi Hashimoto, Kensaku Maejima, Ken Komatsu, Shigeyuki Kakizawa, Yasuyuki Yamaji, Hiroshi Hamamoto, Kenro Oshima, Shigetou Namba

**Affiliations:** Department of Agricultural and Environmental Biology, Graduate School of Agricultural and Life Sciences, The University of Tokyo, Tokyo, Japan; University of Wisconsin-Milwaukee, United States

## Abstract

Culturing is an indispensable technique in microbiological research, and culturing with selective media has played a crucial role in the detection of pathogenic microorganisms and the isolation of commercially useful microorganisms from environmental samples. Although numerous selective media have been developed in empirical studies, unintended microorganisms often grow on such media probably due to the enormous numbers of microorganisms in the environment. Here, we present a novel strategy for designing highly selective media based on two selective agents, a carbon source and antimicrobials. We named our strategy SMART for highly Selective Medium-design Algorithm Restricted by Two constraints. To test whether the SMART method is applicable to a wide range of microorganisms, we developed selective media for *Burkholderia glumae*, *Acidovorax avenae*, *Pectobacterium carotovorum*, *Ralstonia solanacearum*, and *Xanthomonas campestris*. The series of media developed by SMART specifically allowed growth of the targeted bacteria. Because these selective media exhibited high specificity for growth of the target bacteria compared to established selective media, we applied three notable detection technologies: paper-based, flow cytometry-based, and color change-based detection systems for target bacteria species. SMART facilitates not only the development of novel techniques for detecting specific bacteria, but also our understanding of the ecology and epidemiology of the targeted bacteria.

## Introduction

Culture techniques have been indispensable to microbiological research since the 1870s, when they were first established by Louis Pasteur, Robert Koch, and other scientists. The method enables researchers to proliferate and maintain microorganisms stably [1]–[3]. Among the media used for culturing, some possess a degree of selectivity that enables simple, efficient multiplication of a specific microorganism from samples with a large quantity of saprophytes; these are called selective media [4], [5]. Selective media can reliably isolate pathogenic and commercially useful microorganisms. For example, selective media have been used to isolate pathogenic microorganisms in diagnostic medicine and to detect contamination in food or water [4]–[6]. Moreover, selective media are efficient means for growing fastidious microorganisms. Recent research has demonstrated that previously unculturable environmental microorganisms can be grown successfully in a pure culture without any overgrowth of other fast-growing microorganisms [7], [8]. Selective media can recover target microorganisms from environmental samples even if they are slow-growing on a medium. Due to their usefulness, many selective media have been developed for various microorganisms [4], [5]. However, there are no design theories for developing selective media, and each ingredient in selective media has been determined using trial-and-error methods.

Selective media must have two functions: enabling the proliferation of the target microorganism and suppressing unintended microorganisms on the medium. The main challenge is to suppress the growth of saprophytes in analyzed samples [5]. Enormous numbers of microorganisms exist in soil, plant tissues, seawater, and other environments [9]–[14]. For example, the number of species in 1 gram of soil has been variously estimated as approximately 10,000 species [15], 10,000,000 species [16], and 2,000 species [17], [18]. Even with metagenomic analyses, the number of species in a soil community may be so large as to make it impractical to analyze their sequences [19]. Therefore, it seems difficult to culture a target microorganism selectively from among numerous environmental microorganisms. In fact, most reported selective media cannot inhibit the growth of untargeted environmental microorganisms [5].

In this study, we explored how to determine restrictive culture conditions for a target microorganism, *Burkholderia glumae* (*Bgl*). We found that a selective medium could be made using two constraints, and based on this, we established the highly Selective Medium-design Algorithm Restricted by Two constraints (SMART) method. *Bgl* is a causal agent of bacterial grain rot, one of the most problematic diseases of rice [20]. We evaluated the medium developed with the SMART method and compared it to an existing selective medium. Next, we applied the SMART method to four other bacterial species; all of them could be cultured selectively, suggesting the broad utility of the SMART method. We also developed three new highly sensitive detection methods derived from SMART media, and showed the usability of the SMART concept.

## Results

### Comparison of the compositions of reported selective media

The compositions of ten reported selective media are summarized in Table S1, and categorized as 1) natural materials, 2) carbon sources, 3) basal salts, 4) antimicrobials, and 5) colony indicators (Table S1). Most reported selective media contain natural materials (*e*.*g*., peptone and yeast extract). However, synthetic media with no natural materials will suppress untargeted microorganisms better than nutritive media containing natural materials. We also found that media selectivity could be imposed by a carbon source and antimicrobials because most media had the same basal salt composition in common. Therefore, we developed selective media based on non-natural material-derived media containing two selective agents: a carbon source and an antimicrobial. This strategy was named SMART for highly Selective Media-design Algorithm Restricted by Two constraints.

### Determination of the carbon source in SMART

To determine a sole carbon source that the target microorganism *Burkholderia glumae* (*Bgl*) can metabolize, each candidate carbon source listed in Table 1 was added individually to basal synthetic medium (detailed in the Materials and Methods section). The growth of *Bgl* on each medium was tested, and its metabolizable carbon sources were determined (Table 1). We also selected metabolizable carbon sources for *Bgl* based on a metabolic pathway map constructed using genomic information. Online metabolism databases are currently available for a wide range of microorganisms; we used the Kyoto Encyclopedia of Genes and Genomes [21]–[23] or KEGG (http://www.genome.jp/kegg/). PathComp in KEGG is a computational tool that proposes possible reaction pathways between an initial and final compound using information about the presence or absence of known enzymatic reactions. With few exceptions [24], pathogenic bacteria encode a complete gene set for the pentose phosphate pathway, citrate cycle, and glycolysis pathway. Therefore, in this study, a metabolizable carbon source was defined as a substrate whose metabolic pathway links to alpha-d-glucose-6-phosphate, the starting material of the pentose phosphate pathway (Figure S1). The predicted results were in close agreement with the experimental data (Table 1). For example, l-glutamate, glucose, and 20 other substrates were identified as carbon sources metabolizable by *Bgl* using both the computed and experimental methods (Table 1).

**Table 1:** Metabolizable carbon sources of *Burkholderia glumae* using experimental data and genome-based predictions.

Carbon sources	KEGG entry ID	Experimental data by colony formtaion	Prediction by genomic information*
L-glutamate	C00025	+	+
glucose	C00031	+	+
glycine	C00037	-	+
L-lysine	C00047	+	+
L-aspartate	C00049	+	+
L-arginine	C00062	+	+
L-glutamine	C00064	+	+
L-serine	C00065	+	+
L-methionine	C00073	+	+
L-tryptophan	C00078	+	+
L-phenylalanine	C00079	+	+
L-tyrosine	C00082	+	+
sucrose	C00089	+	+
D-fructose	C00095	+	+
L-leucine	C00123	+	+
L-histidine	C00135	-	+
myo-inositol	C00137	-	-
L-proline	C00148	+	+
L-valine	C00183	+	+
cellobiose	C00185	+	+
L-threonine	C00188	+	+
L-sorbose	C00247	+	+
D-mannitol	C00392	-	-
L-isoleucine	C00407	+	+
pectate	C00470	-	-
ribitol	C00474	-	-
D-sorbitol	C00794	+	+
trehalose	C01083	+	+

*Metabolizable carbon sources were predicted using KEGG PathComp.

**Figure 1:**
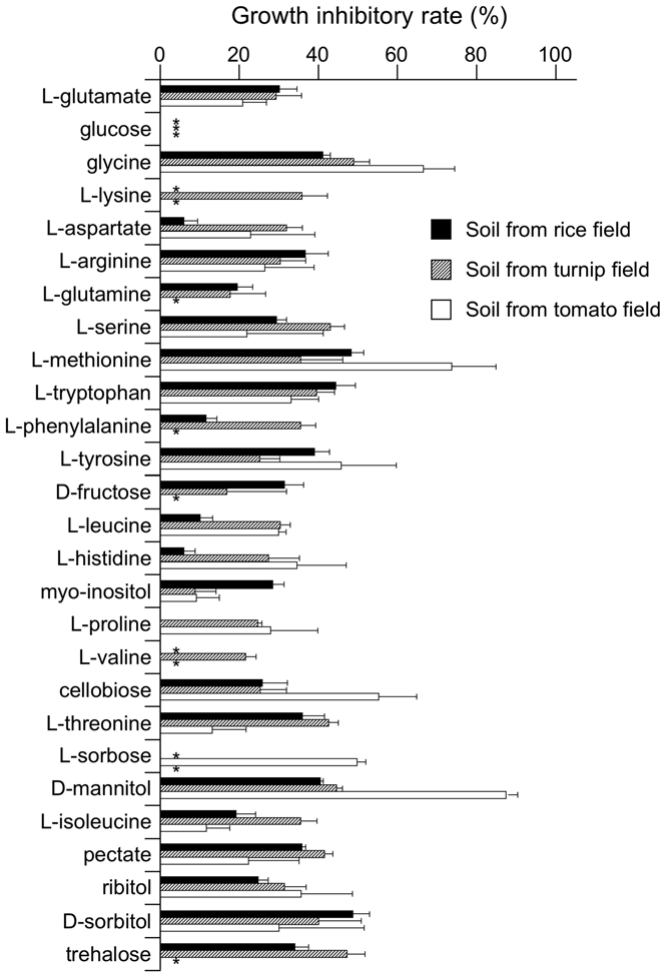
Growth inhibition of soil saprophytes among carbon sources. Each bar indicates the growth inhibitory rate [(1 – the number of colony forming units on each carbon source-added medium/the number on a sucrose-added medium) ×100%]. Asterisks indicate that the saprophyte inhibitory rate was less than that of sucrose.

To choose an optimal carbon source from these candidates, their inhibitory effect on the growth of soil saprophytes was calculated (Figure 1). Soil microorganisms were collected from cultivated soil in a rice field where *Bgl* was isolated in practical tests. The microorganisms were plated on basal synthetic medium supplemented with each carbon source, and the number of saprophyte colonies was counted on each medium. Of the 20 candidate carbon sources, d-sorbitol-supplemented basal medium resulted in the fewest unintended colonies (*i*.*e*., it had the highest rate of saprophyte growth inhibition; Figure 1). Therefore, d-sorbitol was chosen as the optimal carbon source for selective *Bgl* medium. The inhibitory ability of carbon sources against the soil saprophytes in Figure 1 is ranked and summarized in Table S2. The carbon sources used in SMART plays both roles of energy source of the target bacterium and growth inhibitor of saprophytes.

### Determination of resistant antimicrobials in SMART

We selected another selective agent: antimicrobials to which *Bgl* is resistant. d-Sorbitol-containing basal medium was prepared containing each antimicrobial listed in Table 2 individually. The growth of *Bgl* was tested on each medium, and its antimicrobial resistance was determined (Table 2). The antimicrobial resistance of *Bgl* was also predicted from its genomic information. The increasing availability of bacterial genomic sequences enables the prediction of the presence of antimicrobial resistance genes in each genome, based on homology to characterized resistance determinants [25]–[28] deposited in Entrez Gene in the NCBI databases (http://www.ncbi.nlm.nih.gov/). As a result, we identified five candidate antimicrobials for selective *Bgl* medium (Table 2). Experimental data confirmed that *Bgl* strain used in this study was resistant to four out of the five candidates: ampicillin, cephalexin (Sigma), cetrimonium (Nacalai Tesque), and chloramphenicol (Nacalai Tesque) (Table 2) though antibiotics resistance could sometimes differ among bacterial strains. Because the mode of action of cephalexin is similar to that of ampicillin [29], we added only ampicillin, cetrimonium, and chloramphenicol to the selective *Bgl* medium.

**Table 2:** Antimicrobial resistance of *Burkholderia glumae* predicted by the NCBI database compared to experimental data.

Antimicorbial Name	Reported Resistance Gene Name	COG*	Predicted Resistance**	Experimental Data***
ampicillin	beta-lactamase	COG2367V	R	R
	multidrug efflux pump *acrB*	-		
cephalosporine	beta-lactamase	COG2367V	R	R
cetrimonium	quaternary ammoniumcompound resistance protein	COG2076P	R	R
chloramphenicol	chloramphenicolacetyltransferase	-	R	R
	multidrug efflux pump *mdtC*	COG0841V		
gentamicin	aminoglycosidephosphotransferase aac3	-	S	S
	aminoglycosideadenyltransferase aadB	-		
neomycin	aminoglycosidephosphotransferase aac6	-	S	S
penicillin	beta-lactamase	COG2367V	R	S
polymyxin	polymixin resistanceglycosyltransferase	-	S	R
streptomycin	streptomycinphosphotransferase strA	-	S	S
	streptomycinphosphotransferase strB	-		
trimethoprim	dihydrofolate reductase type I	-	S	S
	dihydrofolate reductase type X	-		
gramicidin	hydantoin racemase	-	S	S

*COG stands for clusters of orthologous groups of proteins (http://www.ncbi.nlm.nih.gov/COG/).

**R and S indicate resistant and susceptible, respectively.

***The concentration of antimicrobials added to the medium was 10 ppm.

### Evaluation of a new medium developed by SMART

**Figure 2:**
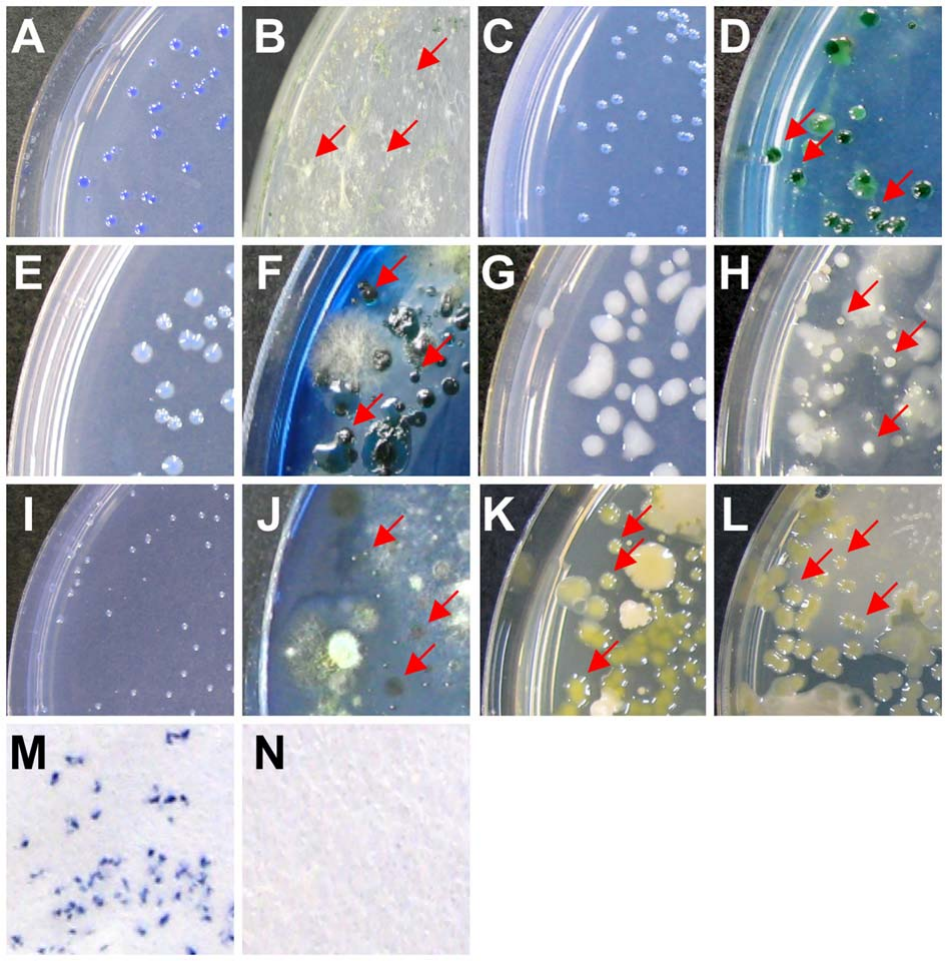
Comparison of colonies formed on selective media produced by SMART and previous methodologies. A suspension of pathogen-inoculated soil was plated on both a SMART medium and an existing selective medium. *Burkholderia glumae* formed colonies on (A) SMART-Glu medium and (B) CCNT medium, as reported by Kawaradani *et al*. [30]. *Acidovorax avenae* subsp. *avenae* formed colonies on (C) SMART-Aav medium and (D) AAC medium, as described by Shirakawa *et al*. [31]. *Pectobacterium carotovorum* formed colonies on (E) SMART-Pca medium and (F) CVP medium, as reported by Cuplles and Kelman [32]. *Ralstonia solanacearum* formed colonies on (G) SMART-Rso medium and (H) SM-1 medium, as shown by Granada and Sequeria [33]. *Xanthomonas campestris* formed colonies on (I) SMART-Xca medium and (J) SM medium, as per Chun *et al*. [34]; (K) on CCA medium, as reported by Mwangi *et al*. [35]; and (L) YTSA-CC medium, as shown by Tripanthi *et al*. [36]. Characteristic colonies of each target bacterium formed, whereas no saprophytes grew on any of the media developed in this study (A, C, E, G, and I). Colonies of the target bacteria are indicated by arrows in B, D, F, H, J, K, and L. On a paper-based selective medium, *B*. *glumae* formed colonies after isolation from diseased soil (M), while none grew from healthy soil (N).

A new selective medium named SMART-Bgl specifically cultured its target bacterium *Bgl*, and the untargeted species tested did not grow on it (Table 3). The composition of the medium is shown in Table S3. Applying the medium to isolate bacteria from diseased soil, only *Bgl* colonies (no other bacteria) were observed on this new medium (Figure 2A). All of the bacteria recovered were identified as *Bgl* using 16S rDNA sequencing analysis. In contrast, unintended saprophytic colonies also grew on the established selective medium [30] (Figure 2B). Therefore, a useful medium can be designed by the SMART method.

**Table 3:** Bacterial strains used in this study and their growth on the selective media.

Species	Strain	Growth on SMART-Bgl	Growth on SMART-Aav	Growth on SMART-Pca	Growth on SMART-Rso	Growth on SMART-Xca
*Burkholderia glumae*	MAFF 301441	+	-	-	-	-
*Acidovorax avenae*	MAFF 301502	-	+	-	-	-
*Agrobacterium rhizogenes*	MAFF 301724	-	-	-	-	-
	MAFF 301725	-	-	-	-	-
*Agrobacterium tumefaciens*	MAFF 301001	-	-	-	-	-
*Burkholderia andropogonis*	Am	-	-	-	-	-
*Pectobacterium carotovorum*	MAFF 301394	-	-	+	-	-
*Pseudomonas cichorii*	u1	-	-	-	-	-
	u2	-	-	-	-	-
*Pseudomonas syringae*	MAFF 301499	-	-	-	-	-
	MAFF 301430	-	-	-	-	-
*Ralstonia solanacearum*	chiba_tomato8945A1	-	-	-	+	-
	kouchi_tomato3-2	-	-	-	+	-
*Xanthomonas campestris*	MAFF 106641	-	-	-	-	+
	MAFF 106644	-	-	-	-	+
	MAFF 211374	-	-	-	-	+
	stock1-1	-	-	-	-	+
	NL 7756	-	-	-	-	+

MAFF; Ministry of Agriculture, Fisheries and Food of Japan.

**Figure 3:**
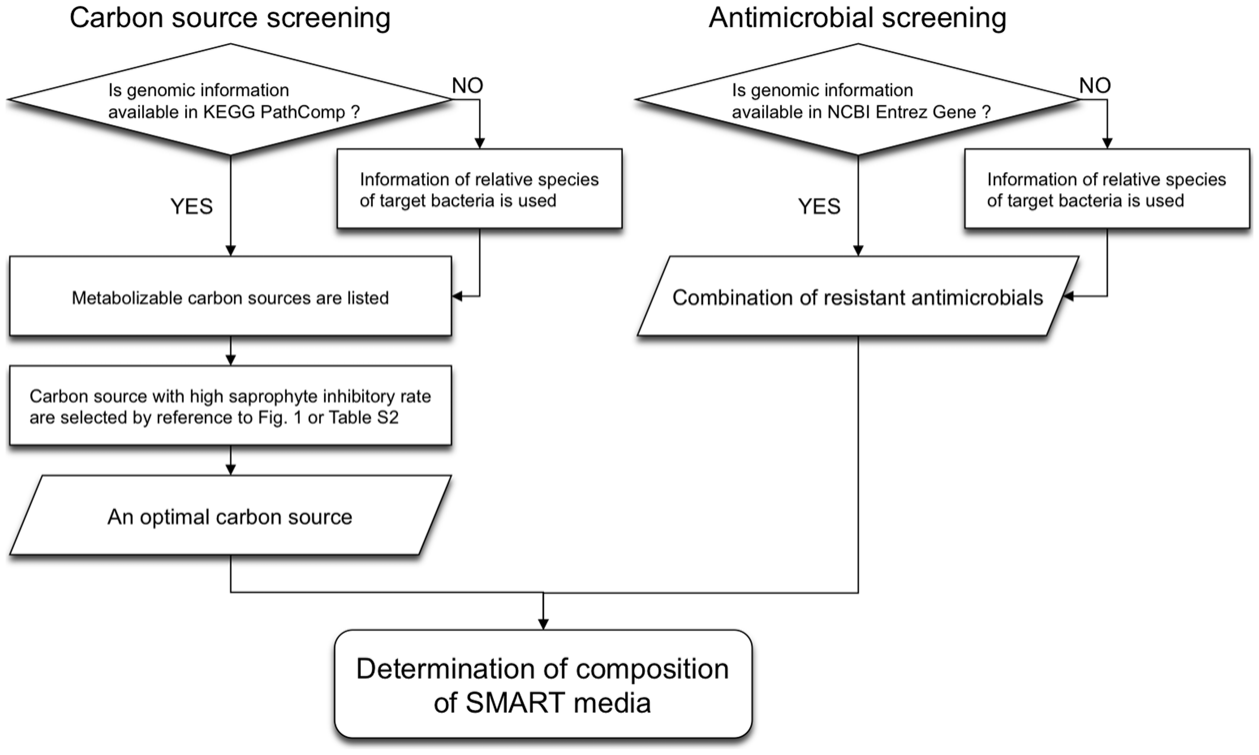
Flowchart of the SMART method. An optimal carbon source and a combination of antimicrobials to which the target bacterium is resistant should be chosen for designing a selective medium using SMART.

In the SMART method (Figure 3), the optimal carbon source for a selective medium is determined in two steps. First, when genomic information on the target bacterium is available in PathComp, metabolizable carbon sources are listed with PathComp. Second, from the listed candidates, the carbon source with the highest saprophyte inhibition rate is chosen by referring to Figure 1 or the recommended carbon source list (Table S2). Antimicrobials to which the target bacterium has possible resistance in NCBI Entrez Gene are used in combination in the medium (Figure 3). When no genomic information on a target bacterium is available in PathComp or NCBI Entrez Gene, information on related species is used to determine the carbon source or antimicrobials.

### Application of SMART to other plant-pathogenic bacteria

To test whether the SMART method is applicable to different bacteria, we applied it to *Acidovorax avenae* subsp. *avenae* (*Aav*), *Pectobacterium carotovorum* subsp. *carotovorum* (*Pca*), *Ralstonia solanacearum* (*Rso*), and *Xanthomonas campestris* pv. *campestris* (*Xca*). Metabolizable carbon sources for these species were predicted using PathComp (Table S4). *Aav*, *Pca*, *Rso*, and *Xca* are pathogens of graminaceous, cruciferous, solanaceous, and cruciferous plants, respectively. From among their candidate metabolizable carbon sources, we selected l-methionine as the carbon source for *Aav* because medium containing l-methionine had the greatest inhibitory effect on the growth of soil saprophytes collected from a rice field (Figure 1). Likewise, we selected trehalose, d-mannitol, and glycine as carbon sources for *Pca*, *Rso*, and *Xca*, respectively. Note that *Pca* could not metabolize glycine, which is the carbon source with the greatest inhibitory effect on saprophytes in the soil of turnip fields, so we chose the second-best candidate.

Antimicrobials for selective media for *Aav*, *Pca*, *Rso*, and *Xca* were selected using the NCBI Entrez Gene database (Table S5). The final compositions are shown in Table S3. In addition to SMART-Bgl for *Bgl*, we developed four other selective media in this study: SMART-Aav for *Aav*, SMART-Pca for *Pca*, SMART-Rso for *Rso*, and SMART-Xca for *Xca*. Each medium was specifically designed to culture its target bacterium (Table 3). In addition, the selective media is not strain-specific because 5 strains of *Xca* (MAFF 106641, MAFF 106644, MAFF 211374, stock1-1 and NL7756) collected from different locations grew on it (Table 3). Colonies of each target bacterium were observed more clearly on the new medium after isolation from diseased soil compared to those on reported selective media [31]–[36] (Figure 2C–L). We confirmed that all of the bacteria recovered with each new selective medium were the target bacteria using 16S rDNA sequencing analysis.

### Establishment of three new detection technologies based on SMART

Each selective medium designed by SMART was highly specific for culturing a target bacterium. This property enabled the application of SMART media to three new detection strategies. First, we substituted filter paper for agar and a Petri dish of plate medium, and developed paper-based selective media, which saves space and cost for bacterial incubation and detection. Inspectors use the paper to detect bacterial contamination of food or soil, saturating it with a suspension of a sample because the paper selectivity inhibits the growth of untargeted microorganisms. After comparing the agar and moisture concentrations during incubation, appropriate conditions for colony growth on paper medium were determined (Table S6). When the specific paper medium was saturated with a suspension of *Bgl*-inoculated soil (6.9×10^3^ cfu/g) and incubated in a plastic bag, bluish-purple colonies of *Bgl* formed on its surface (Figure 2M), whereas none grew when the paper medium was saturated with a suspension of healthy soil (Figure 2N). Similar results were obtained in at least eight independent experiments. Colonies on the surface of the paper medium proliferated when the paper fragment was cut and put in Luria-Bertani broth containing ampicillin (10 mg/L).

The second application of SMART was liquid selective medium (LSM). The compositions of each LSM are shown in Table S3. In 1 mL LSM, one *Bgl* cell multiplied to ca. 10^4^ cells after 24 h incubation (Figure S2 *A*), while the number of saprophytes collected from rice grains decreased to fewer than ten cells after 6 h selective incubation in LSM (Figure S2 *B*). We also developed a new system called SMART-FCM to quantify live specimens of the target bacterium. SMART-FCM is a quantification system that monitors increases in target bacteria using flow cytometry (FCM) after incubation in LSM developed by SMART. To verify the practical application of the proposed SMART-FCM technique, we quantified bacterial cells after selectively incubating four types of sample: one alcohol-sterilized rice grain; five healthy grains; ten artificially *Bgl*-inoculated grains; and ten natural grains possibly infected with *Bgl*. We also performed a plate counting assay with a SMART-Bgl plate. To compare these two techniques, half of each sample was analyzed using SMART-FCM and the other half was analyzed using the plate counting technique. We defined a sample with an FCM count of more than 10^2^ as “positive” (grains infected with the pathogenic bacteria). No false-positive result was obtained with healthy grains and no false-negative was obtained with artificially inoculated grains using either counting technique (Table 4). Positive results were observed for three out of ten natural grains with the FCM analysis, which concurred with the results of the plate counting assay (Table 4). These results indicate that SMART-FCM analysis is as accurate and sensitive as a general plating assay.

**Table 4:** Detection of *Burkholderia glumae* from healthy and infected rice grains using SMART-FCM and a selective medium.

Condition	Sample number	FCM counts**	Counts on a selective plate***
sterilized grain*	1	20	0
healthy grain	2	25	0
	3	15	0
	4	15	0
	5	0	0
	6	5	0
artificially inoculated grain	7	1120	1
	8	1355	1
	9	2350	1
	10	1005	2
	11	5530	2
	12	17280	4
	13	2860	5
	14	4520	5
	15	55445	18
	16	41360	20
possibly infected grain	17	35	0
	18	1495	8
	19	15	0
	20	20	0
	21	5	0
	22	10	0
	23	420	2
	24	10	0
	25	15	0
	26	665	5

* Sterilized with alcohol.

** Microorganisms on rice grains were suspended in 1 mL LSM, and 500 µL LSM was incubated for 24 h and then the bacterial cells were counted using FCM.

*** *B*. *glumae* in the other 500 µL LSM were counted using SMART-Bgl medium.

We also developed a simplified detection method that monitors increases in bacteria using the color change of medium (CCM) supplemented with bromothymol blue. The color of LSM changed from green to yellow when the concentration of a pure *Bgl* culture exceeded 6.4×10^2^ cfu/mL before 24 h incubation (Figure S3A). When analyzing *Bgl*-infected soils ranging from 0 to 68,800 cfu/g soil, the color of the LSM changed when the density of *Bgl* exceeded 1.6×10^3^ cfu/g (equal to 1.6×10^2^ cfu/mL) before 24 h incubation (Figure S3B).

## Discussion

### Magnitude of the SMART approach

Currently, the use of a selective medium is the only way to isolate a target microorganism from a complex environment such as water (sea, river, or lake), soil, or seed surfaces. The major difficulty in developing such media is to suppress the growth of saprophytes in analyzed samples. Unintended microorganisms grow on most reported selective media, which are sometimes referred to as ‘semi-selective’ media [5]. In this study, we established the SMART method, a novel strategy for designing selective media that overcomes this problem. All five selective media developed by SMART were highly specific for the target bacteria, and effectively suppressed the growth of unintended soil saprophytes (Figure 2 and Table 3). Although more than 1,000 species exist in 1 gram of soil [15]–[18], SMART media suppressed these numerous saprophytes and grew the target species selectively. The selectivity was surprisingly high, even compared to previously reported selective media [30]–[36]. The breakthrough in SMART is the concept of using two non-natural selective agents: a carbon source and antimicrobials. A previous concept developed a selective medium by adding antimicrobials to a natural material-derived nutritive medium [4], [5] because it is time-consuming to determine an appropriate synthetic minimal medium for the growth of a target bacterium. Due to the lack of selectivity, unintended microorganisms usually grow on this type of medium, even after it is supplemented with several antimicrobials. In contrast, SMART provided the optimal path for determining the composition of a selective medium.

In this study, we established the procedure for SMART, comparing genome-based predictions with experimental data on *Burkholderia glumae* (*Bgl*), and subsequently applied it to four other phytopathogenic bacteria. We did not examine SMART on Gram-positive bacteria in this study because most phytopathogenic bacteria are Gram-negative. However, since SMART functioned for all the five species tested, it should be applicable to other bacteria, such as Gram-positive animal pathogens. From a commercial perspective, SMART will enable the development of isolation media for lactic acid bacteria, photosynthetic bacteria, and other “effective microorganisms” (so-called EM) used for wastewater treatment [37]. SMART media can recover these useful bacteria from environmental samples even if they are masked by an overwhelming growth of saprophytes on regular media.

### Applicability of genomic information to SMART

In the SMART method, there is no need to test all possible combinations of carbon sources and antimicrobials manually to develop a synthetic selective medium. Genomic information can be used to select both the metabolizable carbon source and the appropriate antimicrobials. Because genomic information is accumulating rapidly with recent progress in DNA sequencing technology [38], [39], such information is increasingly available to researchers. The predictions concerning these two factors were highly consistent with the experimental data in this study (Tables 1, 2, S4, and S5). The disagreements between predicted and experimental data could be classified into four patterns: (a) predicted metabolizable carbon sources are non-metabolizable in practice (*e*.*g*., glycine and l-histidine for *Bgl* in Table 1); (b) predicted non-metabolizable carbon sources are metabolizable in practice (*e*.*g*., sucrose and l-sorbose for *Acidovorax avenae* in Table S4); (c) the species is susceptible to antimicrobials it is predicted to be resistant to (*e*.*g*., penicillin for *Bgl* in Table 2); and (d) the species is resistant to antimicrobials it is predicted to be susceptible to (*e*.*g*., polymyxin). In case (a), the disagreement probably resulted from a metabolic pathway that is irreversible *in vivo*, even if it is predicted to be a reversible pathway *in silico* [21]–[23]. In contrast, (b) involves a case in which the bypassing pathway from carbon sources to the pentose phosphate pathway and citrate cycle is still unknown [40]. In case (c), the minimum inhibitory concentration of an antimicrobial was below 10 ppm, which was the concentration of antimicrobials added to the medium in this study, although the target bacterium has slight resistance to the antimicrobial. Finally, in case (d), the disagreement might have been caused by the presence of unknown broad-range resistance mechanisms, such as multidrug efflux pumps [41]. In screening carbon sources and antimicrobials, cases (a) and (c) are not problematic because they do not result in overlooking usable substrates. In contrast, usable candidate carbon sources and antimicrobials are overlooked in cases (b) and (d), although only a few such cases were observed in this study. Overall, the results indicate that the optimal carbon source and antimicrobials for highly selective media can be determined when researchers examine two or three candidates based on genomic information.

### Three notable detection techniques derived from SMART

We also demonstrated the applicability of SMART to developing new detection systems based on its extreme specificity. Using a paper-based selective medium and CCM, inspectors in the field can detect the presence of a target bacterium without a clean bench or other special equipment. In addition, these two methods require much less space to incubate and maintain bacteria than conventional plate media. In terms of the detection limit, SMART-FCM and CCM can detect one cell of a pathogenic bacterium per seed lot and 1.6×10^3^ cells per gram of soil within 24 hours, respectively (Table 4 and Figure S3). Specific PCR or serological techniques are routinely used to inspect food and water [4]–[6]. The sensitivity of SMART-FCM and CCM for detecting microorganisms from environmental samples was much greater than that of the PCR or ELISA methods, which are about 10^4^ and 10^5^ cells per sample, respectively [5], [42], [43]. Like all paper-based media, SMART-FCM and CCM are also easy to use. Advanced DNA-based methods have not yet entirely replaced traditional culture tests in diagnostic laboratories because trained personnel are needed for DNA extraction, electrophoresis, and other procedures [5], [44]. Because the SMART-derived detection methods do not require complicated procedures, there is no need for trained personnel.

To detect target microorganisms, techniques using FCM, CCM, and paper-based media would be impossible without SMART. Open-air handling of the detection methods is realized because of the SMART-driven high suppression of environmental saprophytes. Selective media and derived techniques are useful for agriculture, microbiology, clinical science, food inspection, and other fields. The greatest advantage of the SMART medium-design technique is its general applicability to a wide range of bacteria unless they are naturally auxotrophic. We believe that SMART theory will facilitate not only the development of novel techniques for detecting specific bacteria, but also our understanding of the ecology and epidemiology of bacteria.

## Materials and Methods

### Carbon source screening using the KEGG database

Metabolizable carbon sources were predicted using PathComp in the KEGG database (http://www.genome.jp/kegg/). After a target bacterium was selected (“search against”), the “initial compound” in Table 1 was inputted. Alpha-d-glucose 6-phosphate (compound ID C00668) was selected as the final compound (cutoff length 20).

### Antimicrobial screening using the NCBI database

Resistant antimicrobials were predicted using Entrez Gene in the NCBI genes database (http://www.ncbi.nlm.nih.gov/). Antimicrobial resistance genes can be explored by entering “organism name[ORGN] resistance gene name” (*e*.*g*., “*Burkholderia glumae*[ORGN] beta-lactamase”) in the search box.

### Preparing medium

The compositions of the selective media for all of the bacteria tested are shown in Table S3. All chemicals used for the media were purchased from Wako Pure Chemical Industries, unless otherwise indicated. To prepare selective plates, 15 g agar was added to a basal salt solution containing Na_2_HPO_4_, KH_2_PO_4_, NH_4_Cl, MgSO_4_, and FeSO_4_ and autoclaved at 120°C for 20 min. After the solution was cooled to 55°C, 10 mL selective solution containing the carbon source and antimicrobials was filter-sterilized and then added to the basal medium. To prepare a paper-based medium, filter paper (Advantec 5B) was saturated with the basal medium mixed with selective solution, and air-dried. To prepare LSM, a selective solution was added to the basal medium and filter-sterilized. Bromothymol blue was added to the LSM at a final concentration of 200 mg/L for diagnosis using CCM.

### Soil supernatant

Soil samples were collected at a depth of 10 cm from rhizospheric soil in turnip, tomato, and rice fields in Tokyo, Japan. Bacteria were extracted from each soil sample using the following procedure: 1 g soil was suspended in 10 mL distilled water in a 50 mL centrifuge tube (BD Biosciences) and vortexed for 10 min. After stationary incubation for 10 min at room temperature, the supernatant was used to evaluate the medium as a source of target bacterium and soil saprophytic bacteria.

### Identification of bacteria by 16S rDNA sequencing

16S rDNA was amplified using universal primers 8f (5′-AGAGT TTGAT CCTGG CTCAG-3′) and 1492r (5′-GGTTA CCTTG TTACGA CTT-3′). Colony-PCR was performed using the following protocol: initial denaturation at 96°C for 5 min, followed by 25 cycles of 96°C for 1 min, 52°C for 40 s, and 72°C for 3 min, with a final extension at 72°C for 7 min. Each 25-µL PCR reaction consisted of 0.25 µL rTaq DNA polymerase (TaKaRa), primers (5.0 µM each), and PCR buffer. PCR products were sequenced using BigDye Terminator v3.1 Cycle Sequencing Kit (ABI) with primers 8f or 1492r. Sequences were determined with a 3130xl genetic analyzer (ABI). Forward and reverse sequences were assembled using Seqman (DNAStar), and the consensus sequences were obtained. The species were determined by identifying those with the highest similarity to the consensus sequence.

### Calculation of the growth inhibition rate

Soil supernatant was spread on each carbon source-supplemented basal medium (Table 1). The number of colonies on each medium was counted after 5 days of incubation at 30°C. The growth inhibition rate was calculated as (1 – the number of colonies on each carbon source-added medium/the number on sucrose-supplemented medium) ×100%.

### Incubation conditions of paper-based medium

A paper-based medium (2×3.5 cm) was saturated with soil supernatant, and air-dried so that the bacteria adhered firmly to the filaments of the filter paper. The paper was placed in a plastic bag (7×10 cm) and 100 µL distilled water was added to keep the paper surface moist. After a 3-day incubation at 30°C, colonies formed on the surface.

### Bacterial cell counting with a flow cytometer

To enumerate the target bacterium in LSM, 100 µL LSM was mixed with 1 µL ChemChrome V23 (AES Chemunex, France), and the volume was increased to 1 mL with water. ChemChrome V23 reacts only with live cells. These samples were analyzed using a CyFlow (Partec, Germany) equipped with a 20 mm blue solid-state laser operating at 488 nm. The green fluorescence emission (FL1) of ChemChrome V23 (FCM count) was measured using a band-pass filter at 525 nm (510–540 nm).

### Growth curves of *B*. *glumae* and saprophytes in LSM

The fluctuation in the density of *Bgl* in LSM was measured as follows. A small amount of *Bgl* (fewer than 20 cells) was incubated with 1 mL LSM in a 1.5 mL tube. During 12 h incubation, 100 µL LSM-incubating *Bgl* was collected every 3 h (including a starting point sample) and analyzed with CyFlow to count the number of *Bgl*. The number of *Bgl* at each incubation period was divided by that at 0 h, and a growth curve per *Bgl* cell in the LSM was plotted. The density fluctuation of saprophytes incubated in LSM was recorded as follows. A rice grain was used as a source of saprophytes because *Bgl* is a rice grain pathogen. The microorganisms on the surface of a healthy rice grain were suspended in 1.5 mL tubes containing 1 mL LSM using sonic disintegration. After removing the rice grain, the LSM was incubated at 37°C. The saprophyte density was measured using CyFlow 0, 3, 12, and 24 h after incubation, and a growth curve for the saprophytes in LSM was obtained.

### Detection of plant-pathogenic bacteria from infected rice grains

To confirm the practical use of SMART-FCM, *Bgl* was detected from rice grains. The microorganisms on rice grains were suspended in 1 mL LSM using sonic disintegration. To compare SMART-FCM to a plating method, half (500 µL) of the LSM was incubated for 24 h, followed by FCM analysis. The other half of the LSM (without incubation) was centrifuged at 5000×*g* for 5 min at room temperature. After removing 400 µL supernatant, the pellet was resuspended and plated on a SMART-Bgl plate.

## Supporting Information

Figure S1 **Part of the metabolic pathway map of** ***Burkholderia glumae*** **.** Reaction enzymes that *B*. *glumae* encodes and does not encode are denoted by blue and red arrows, respectively. *B*. *glumae* has a pathway from cellobiose, trehalose, and d-fructose, but not from pectate to alpha-d-glucose-6-phosphate. Therefore, an *a priori* methodology predicts that cellobiose, trehalose, and d-fructose are metabolizable carbon sources for *B*. *glumae*, while pectate is not.
(TIF)

Figure S2 **Selective growth of** ***Burkholderia glumae*** **and the repression of rice seed saprophytes in LSM.** *B*. *glumae* and saprophytes from rice grains were incubated in LSM and their numbers were counted by FCM every 3 h. One cell of *B*. *glumae* multiplied to approximately 10^4^ cfu in LSM after 24 h. In contrast, the number of saprophytes from a rice grain decreased to below 10 FCM counts/mL after 6 h incubation.
(TIF)

Figure S3 **Soil diagnosis of** ***Burkholderia glumae*** **infection using the CCM method.** (A) *B*. *glumae* pure culture was added to 1 mL LSM and the change in the color of the medium was observed after 24 h incubation. The color changed from green to yellow when the number of *B*. *glumae* exceeded 6.4×10^2^ cfu/mL before incubation. (B) Samples (0.01 g) of soil were incubated in 1 mL LSM for 24 h. The number of *B*. *glumae* in each soil sample was counted using SMART-Bgl medium before incubation. The color of LSM changed from green to yellow when the density of *B*. *glumae* exceeded 2.6×10^3^ cfu/g (equal to 26 cfu/1 mL LSM) before incubation.
(TIF)

Table S1 **Comparison of the compositions of reported selective media.**
(DOC)

Table S2 **Recommended carbon source list.**
(DOC)

Table S3 **Compositions of the selective media developed in this study.**
(DOC)

Table S4 **Metabolizable carbon sources of four target bacteria based on experimental data and genome-based predictions.**
(DOC)

Table S5 **Antimicrobial resistance predicted by the NCBI database compared to experimental data.**
(DOC)

Table S6 **Determination of the appropriate agar and moisture concentration for colony formation on paper-based medium.**
(DOC)
