# Supplementary figures and images for: New Detection Systems of Bacteria Using Highly Selective Media Designed by SMART: Selective Medium-Design Algorithm Restricted by Two Constraints
Source: PLoS One. 2011 Jan 27;6(1):e16512. doi: 10.1371/journal.pone.0016512 (PMC3029383; doi:10.1371/journal.pone.0016512)

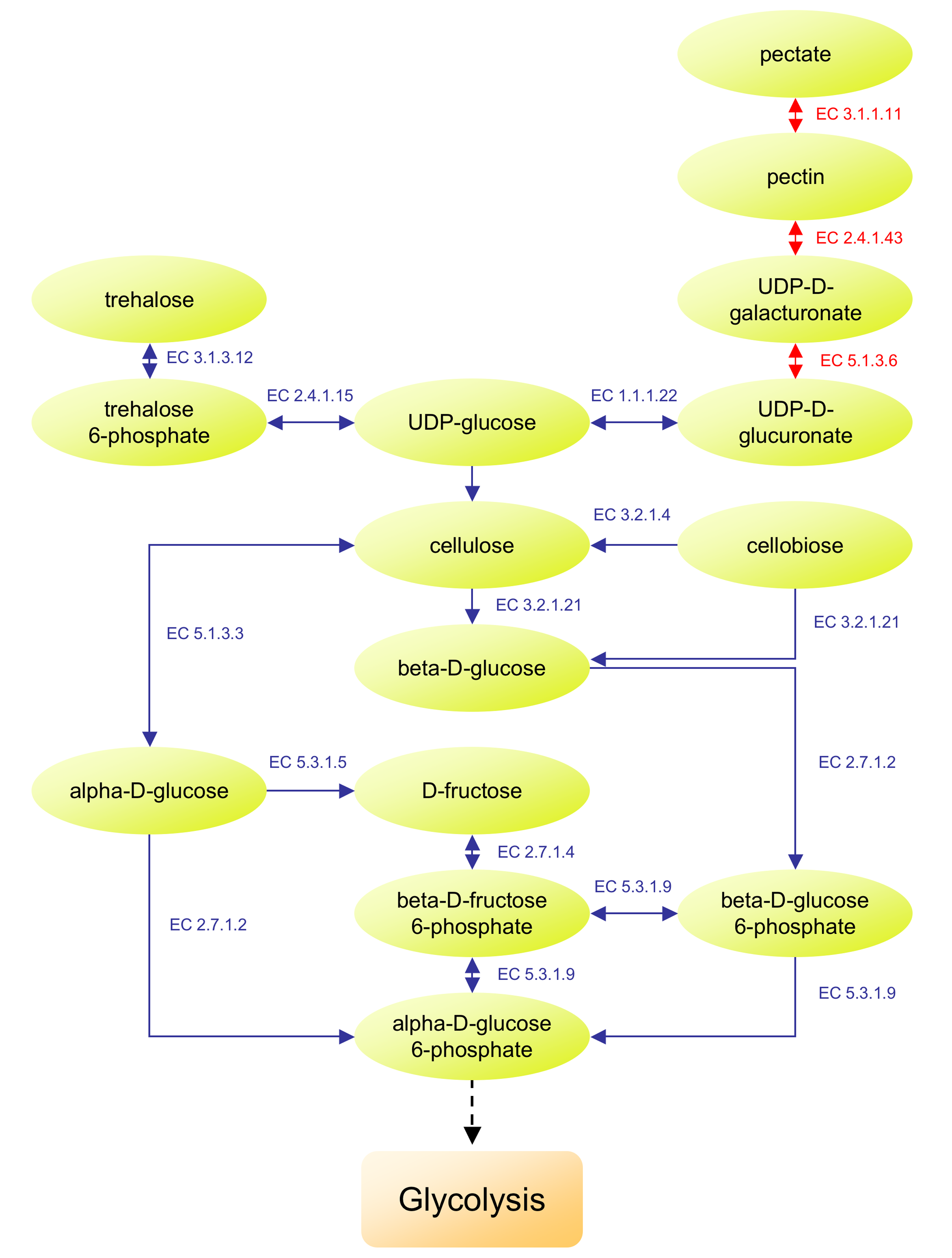

Supplement: Figure S1 — Part of the metabolic pathway map of Burkholderia glumae . Reaction enzymes that B. glumae encodes and does not encode are denoted by blue and red arrows, respectively. B. glumae has a pathway from cellobiose, trehalose, and d-fructose, but not from pectate to alpha-d-glucose-6-phosphate. Therefore, an a priori methodology predicts that cellobiose, trehalose, and d-fructose are metabolizable carbon sources for B. glumae, while pectate is not. (TIF) [file pone.0016512.s001.tif]

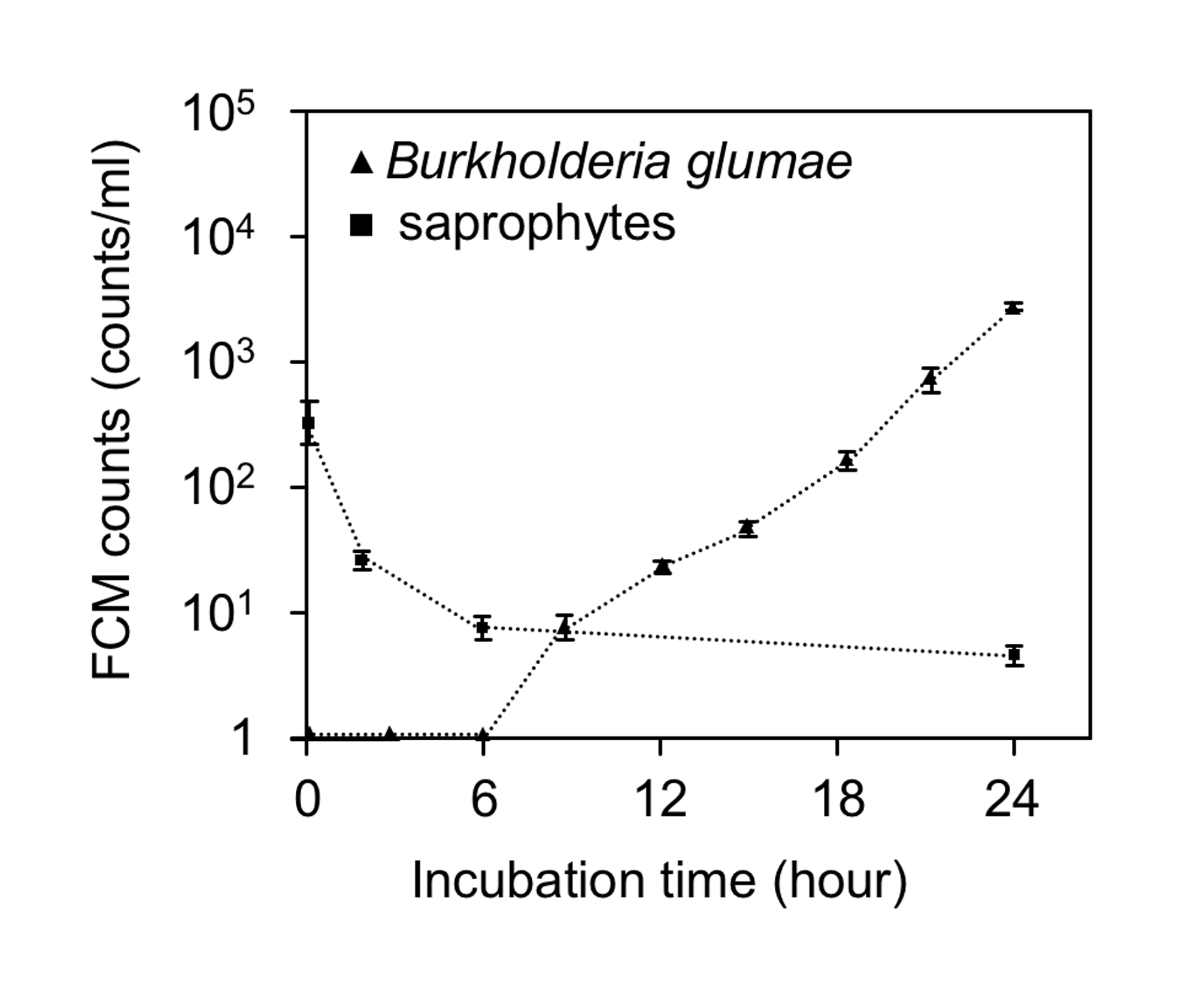

Supplement: Figure S2 — Selective growth of Burkholderia glumae and the repression of rice seed saprophytes in LSM. B. glumae and saprophytes from rice grains were incubated in LSM and their numbers were counted by FCM every 3 h. One cell of B. glumae multiplied to approximately 104 cfu in LSM after 24 h. In contrast, the number of saprophytes from a rice grain decreased to below 10 FCM counts/mL after 6 h incubation. (TIF) [file pone.0016512.s002.tif]

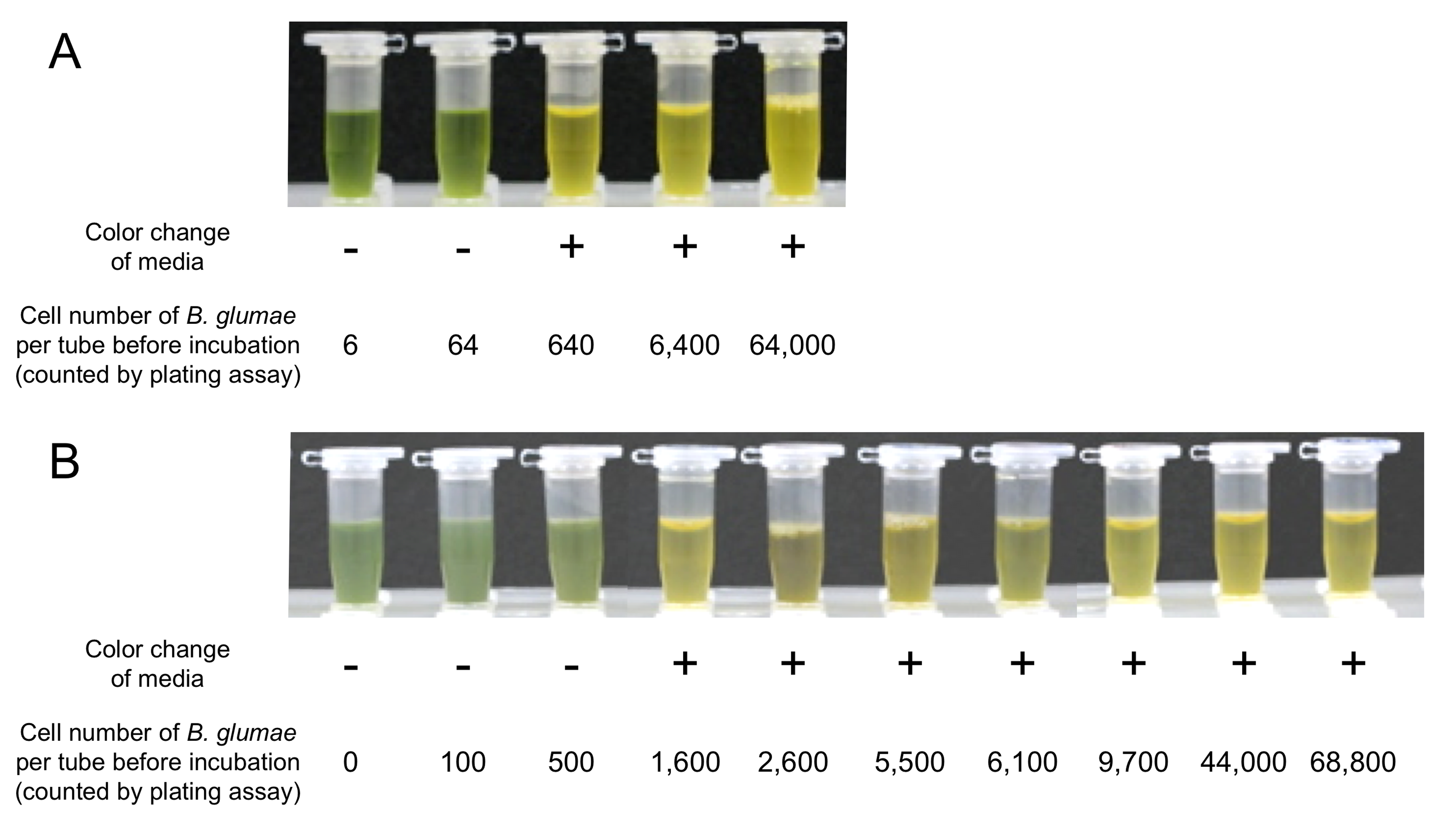

Supplement: Figure S3 — Soil diagnosis of Burkholderia glumae infection using the CCM method. (A) B. glumae pure culture was added to 1 mL LSM and the change in the color of the medium was observed after 24 h incubation. The color changed from green to yellow when the number of B. glumae exceeded 6.4×10^2^ cfu/mL before incubation. (B) Samples (0.01 g) of soil were incubated in 1 mL LSM for 24 h. The number of B. glumae in each soil sample was counted using SMART-Bgl medium before incubation. The color of LSM changed from green to yellow when the density of B. glumae exceeded 2.6×10^3^ cfu/g (equal to 26 cfu/1 mL LSM) before incubation. (TIF) [file pone.0016512.s003.tif]
