# Supplementary material for: New Detection Systems of Bacteria Using Highly Selective Media Designed by SMART: Selective Medium-Design Algorithm Restricted by Two Constraints
Source: PLoS One. 2011 Jan 27;6(1):e16512. doi: 10.1371/journal.pone.0016512 (PMC3029383; doi:10.1371/journal.pone.0016512)
Supplement: Table S1 — Comparison of the compositions of reported selective media. (DOC) [file pone.0016512.s004.doc]

**Table S1**. Comparison of the compositions of reported selective media.

| target bacteria | *Burkholderia glumae* |  | *Acidovorax avenae* |  | *Pectobacterium carotovorum* | | |
| --- | --- | --- | --- | --- | --- | --- | --- |
| selective medium | CCNT medium |  | AAC medium |  | D medium | CVP medium | PEC-YA medium |
|  | Kawaradani et al. |  | Shirakawa et al. |  | Kado and Heskett | Cuplles and Kelman | Starr et al. |
|  | 2000 |  | 2000 |  | 1970 | 1974 | 1977 |
| natural materials | peptone |  | yeast extract |  | casein hydrolysate |  | yeast extract |
|  | yeast extract |  |  |  |  |  |  |
|  |  |  |  |  |  |  |  |
| carbon source | inositol |  | adipic acid |  | arabinose | citrate | pectate |
|  |  |  |  |  | glycine | pectate |  |
|  |  |  |  |  | sucrose |  |  |
| basal salts^*^ |  |  | NH4SO4 |  | LiCl | CaCl2 |  |
|  |  |  | KH2PO4 |  | NaCl |  |  |
|  |  |  | Na2HPO4 |  | MgSO4 |  |  |
|  |  |  | MgSO4 |  |  |  |  |
|  |  |  | Na2MoO4 |  |  |  |  |
| antimicrobial | ceramide |  | ampicillin |  | SDS^****^ | SDS |  |
|  | chloramphenicol |  | cycloheximide |  |  |  |  |
|  | novobiocin |  | novobiocin |  |  |  |  |
|  | TPN^**^ |  | phenethicillin |  |  |  |  |
|  |  |  |  |  |  |  |  |
| colony indicator |  |  | BTB^***^ |  | acid fuchsin | crystal violet | BTB |
|  |  |  |  |  | BTB |  |  |

**Table S1. (Continued)**

| target bacteria |  | *Xanthomonoas campestris* | | |  | *Ralstonia solanacearum* | |
| --- | --- | --- | --- | --- | --- | --- | --- |
| selective medium |  | SM medium | CCA medium | YTSA-CC medium |  | FSM medium | SM-1 medium |
|  |  | Chun and Alvarez | Mwangi et al. | Tripathi et al. |  | Nesmith and Jenkins | Granada and Sequeira |
|  |  | 1983 | 2007 | 2007 |  | 1979 | 1983 |
| natural materials |  | potato starch | beef extract | tryptone |  | casein hydrolysate | casein hydrolysate |
|  |  |  | peptone | yeast extract |  | peptone | peptone |
|  |  |  | yeast extract |  |  | yeast extract |  |
| carbon source |  | glucose | cellobiose | sucrose |  | citrate | glucose |
|  |  | methionine | glucose |  |  | glucose |  |
|  |  |  |  |  |  |  |  |
| basal salts^a^ |  | NH4Cl | NH4Cl |  |  | MgSO4 |  |
|  |  | KH2PO4 | K2HPO4 |  |  | FeC6H5O7 |  |
|  |  | Na2HPO4 | MgSO4 |  |  | MnSO4 |  |
|  |  | MgSO4 |  |  |  | ZnSO4 |  |
|  |  | and others |  |  |  | and others |  |
| antimicrobial |  | cycloheximide | cephalexin | cephalexin |  | benomyl | chlorothalonil |
|  |  |  | cycloheximide | cycloheximide |  | chloroneb | cycloheximide |
|  |  |  | fluorouracil |  |  | cycloheximide | polymyxin |
|  |  |  |  |  |  | dichloran | thimerosal |
|  |  |  |  |  |  | and others | and others |
| colony indicator |  | TZC^*****^ |  |  |  | TZC | crystal violet |
|  |  |  |  |  |  |  | TZC |

* including sources of nitrogen, sulfur, phosphorus, and minerals

** tetrachloroisophthalonitrile

*** bromothymol blue

**** sodium dodecyl sulfate

***** tetrazolium chloride
