# Supplementary material for: New Detection Systems of Bacteria Using Highly Selective Media Designed by SMART: Selective Medium-Design Algorithm Restricted by Two Constraints
Source: PLoS One. 2011 Jan 27;6(1):e16512. doi: 10.1371/journal.pone.0016512 (PMC3029383; doi:10.1371/journal.pone.0016512)
Supplement: Table S2 — Recommended carbon source list. (DOC) [file pone.0016512.s005.doc]

**Table S2**. Recommended carbon source list.

| Carbon sources | Growth inhibition ranking^*^ against soil saprophytes of | | |
| --- | --- | --- | --- |
|  | rice | turnip | tomato |
| glycine | 4 | 1 | 3 |
| L-methionine | 1 | 10 | 2 |
| D-mannitol | 5 | 3 | 1 |
| D-sorbitol | 2 | 7 | 10 |
| trehalose | 10 | 2 | 21 |
| L-serine | 13 | 4 | 16 |
| L-tryptophan | 3 | 8 | 9 |
| L-tyrosine | 6 | 18 | 6 |
| cellobiose | 14 | 20 | 4 |
| ribitol | 16 | 14 | 7 |
| L-glutamate | 12 | 17 | 17 |
| L-arginine | 7 | 15 | 13 |
| L-leucine | 20 | 16 | 11 |
| L-histidine | 22 | 19 | 8 |
| L-threonine | 8 | 5 | 18 |
| pectate | 9 | 6 | 15 |
| L-aspartate | 21 | 13 | 14 |
| D-fructose | 11 | 24 | 21 |
| myo-inositol | 15 | 25 | 20 |
| L-proline | 23 | 21 | 12 |
| L-sorbose | 23 | 26 | 5 |
| L-isoleucine | 18 | 12 | 19 |
| glucose | 23 | 26 | 21 |
| L-lysine | 23 | 9 | 21 |
| L-glutamine | 17 | 23 | 21 |
| L-phenylalanine | 19 | 11 | 21 |
| sucrose | 23 | 26 | 21 |
| L-valine | 23 | 22 | 21 |

* Carbon sources are arrayed in order of recommendation from the top of the list, based on the growth inhibitory rates of rice, turnip, and tomato soils in Figure 1.
