# Supplementary material for: New Detection Systems of Bacteria Using Highly Selective Media Designed by SMART: Selective Medium-Design Algorithm Restricted by Two Constraints
Source: PLoS One. 2011 Jan 27;6(1):e16512. doi: 10.1371/journal.pone.0016512 (PMC3029383; doi:10.1371/journal.pone.0016512)
Supplement: Table S3 — Compositions of the selective media developed in this study. (DOC) [file pone.0016512.s06.doc]

**Table S3**. Compositions of the selective media developed in this study.

| SMART-Bgl medium plate | |  | SMART-Aac medium plate | |  | SMART-Pca medium plate | |  | SMART-Rso medium plate | |  | SMART-Xca medium plate | |
| --- | --- | --- | --- | --- | --- | --- | --- | --- | --- | --- | --- | --- | --- |
| For *Burkholderia glumae* | |  | For *Acidovorax avenae* | |  | For *Pectobacterium carotovorum* | |  | For *Ralstonia solanacearum* | |  | For *Xanthomonas campestris* | |
| D-sorbitol | 1 g |  | L-methionine | 1 g |  | Trehalose | 1 g |  | D-Mannitol | 1 g |  | Glycine | 1 g |
| Na2HPO4 | 3 g |  | Na2HPO4 | 3 g |  | Na2HPO4 | 3 g |  | Na2HPO4 | 3 g |  | Na2HPO4 | 3 g |
| KH2PO4 | 3 g |  | KH2PO4 | 3 g |  | KH2PO4 | 3 g |  | KH2PO4 | 3 g |  | KH2PO4 | 3 g |
| NH4Cl | 1 g |  | NH4Cl | 1 g |  | NH4Cl | 1 g |  | NH4Cl | 1 g |  | NH4Cl | 1 g |
| MgSO4 | 0.25 g |  | MgSO4 | 0.25 g |  | MgSO4 | 0.25 g |  | MgSO4 | 0.25 g |  | MgSO4 | 0.25 g |
| FeSO4 | 5 mg |  | FeSO4 | 5 mg |  | FeSO4 | 5 mg |  | FeSO4 | 5 mg |  | FeSO4 | 5 mg |
| Crystal violet | 3 mg |  | Crystal violet | 3 mg |  | Crystal violet | 3 mg |  | Crystal violet | 3 mg |  | Crystal violet | 3 mg |
| Agar | 15 g |  | Agar | 15 g |  | Agar | 15 g |  | Agar | 15 g |  | Agar | 15 g |
| DW | 1 L |  | DW | 1 L |  | DW | 1 L |  | DW | 1 L |  | DW | 1 L |
| Cycloheximide | 50 mg |  | Cycloheximide | 50 mg |  | Cycloheximide | 50 mg |  | Cycloheximide | 50 mg |  | Cycloheximide | 50 mg |
| Ampicillin | 10 mg |  | Ampicillin | 10 mg |  | Cetrimonium | 10 mg |  | Chloramphenicol | 10 mg |  | Cepharexin | 10 mg |
| Cetrimonium | 10 mg |  | Cetrimonium | 10 mg |  | Tyrothricin | 10 mg |  | Polymixin | 10 mg |  | Penicillin | 10 mg |
| Chloramphenicol | 10 mg |  | Polymixin | 10 mg |  |  |  |  |  |  |  |  |  |

**Table S2**. (Continued)

| SMART-Bgl LSM | |  | SMART-Aac LSM | |  | SMART-Pca LSM | |  | SMART-Rso LSM | |  | SMART-Xca LSM | |
| --- | --- | --- | --- | --- | --- | --- | --- | --- | --- | --- | --- | --- | --- |
| For *Burkholderia glumae* | |  | For *Acidovorax avenae* | |  | For *Pectobacterium carotovorum* | |  | For *Ralstonia solanacearum* | |  | For *Xanthomonas campestris* | |
| D-sorbitol | 1 g |  | L-methionine | 1 g |  | Trehalose | 1 g |  | Mannitol | 1 g |  | Glycine | 1 g |
| Na2HPO4 | 3 g |  | Na2HPO4 | 3 g |  | Na2HPO4 | 3 g |  | Na2HPO4 | 3 g |  | Na2HPO4 | 3 g |
| KH2PO4 | 3 g |  | KH2PO4 | 3 g |  | KH2PO4 | 3 g |  | KH2PO4 | 3 g |  | KH2PO4 | 3 g |
| NH4Cl | 1 g |  | NH4Cl | 1 g |  | NH4Cl | 1 g |  | NH4Cl | 1 g |  | NH4Cl | 1 g |
| MgSO4 | 0.25 g |  | MgSO4 | 0.25 g |  | MgSO4 | 0.25 g |  | MgSO4 | 0.25 g |  | MgSO4 | 0.25 g |
| FeSO4 | 5 mg |  | FeSO4 | 5 mg |  | FeSO4 | 5 mg |  | FeSO4 | 5 mg |  | FeSO4 | 5 mg |
| Crystal violet | 3 mg |  | Crystal violet | 3 mg |  | Crystal violet | 3 mg |  | Crystal violet | 3 mg |  | Crystal violet | 3 mg |
| DW | 1 L |  | DW | 1 L |  | DW | 1 L |  | DW | 1 L |  | DW | 1 L |
| Cycloheximide | 5 mg |  | Cycloheximide | 5 mg |  | Cycloheximide | 5 mg |  | Cycloheximide | 5 mg |  | Cycloheximide | 5 mg |
| Ampicillin | 1 mg |  | Ampicillin | 1 mg |  | Cetrimide | 1 mg |  | Chloramphenicol | 1 mg |  | Cepahrexin | 1 mg |
| Cetrimonium | 1 mg |  | Cetrimide | 1 mg |  | Tryothricin | 1 mg |  | Polymixin | 1 mg |  | Penicillin | 1 mg |
| Chloramphenicol | 1 mg |  | Polymixin | 1 mg |  |  |  |  |  |  |  |  |  |
