# Supplementary material for: New Detection Systems of Bacteria Using Highly Selective Media Designed by SMART: Selective Medium-Design Algorithm Restricted by Two Constraints
Source: PLoS One. 2011 Jan 27;6(1):e16512. doi: 10.1371/journal.pone.0016512 (PMC3029383; doi:10.1371/journal.pone.0016512)
Supplement: Table S4 — Metabolizable carbon sources of four target bacteria based on experimental data and genome-based predictions. (DOC) [file pone.0016512.s007.doc]

**Table S4**. Metabolizable carbon sources of four target bacteria based on experimental data and genome-based predictions.

| Carbon sources | KEGG |  | *Acidovorax avenae* | |  | *Pectobacterium carotovorum* | |
| --- | --- | --- | --- | --- | --- | --- | --- |
|  | entry ID |  | experimental | predicted* |  | experimental | predicted |
| L-glutamate | C00025 |  | + | + |  | + | + |
| glucose | C00031 |  | + | + |  | + | + |
| glycine | C00037 |  | + | + |  | - | + |
| L-lysine | C00047 |  | + | + |  | + | + |
| L-aspartate | C00049 |  | + | + |  | + | + |
| L-arginine | C00062 |  | - | + |  | - | + |
| L-glutamine | C00064 |  | + | + |  | - | + |
| L-serine | C00065 |  | + | + |  | + | + |
| L-methionine | C00073 |  | + | + |  | + | + |
| L-tryptophan | C00078 |  | - | + |  | - | + |
| L-phenylalanine | C00079 |  | + | + |  | + | + |
| L-tyrosine | C00082 |  | + | + |  | + | + |
| sucrose | C00089 |  | + | - |  | + | + |
| D-fructose | C00095 |  | + | + |  | + | + |
| L-leucine | C00123 |  | + | + |  | - | - |
| L-histidine | C00135 |  | + | + |  | + | + |
| myo-inositol | C00137 |  | - | - |  | - | - |
| L-proline | C00148 |  | + | + |  | + | + |
| L-valine | C00183 |  | + | + |  | + | + |
| cellobiose | C00185 |  | - | - |  | + | + |
| L-threonine | C00188 |  | + | + |  | + | + |
| L-sorbose | C00247 |  | + | - |  | + | - |
| D-mannitol | C00392 |  | - | - |  | + | + |
| L-isoleucine | C00407 |  | - | + |  | + | + |
| pectate | C00470 |  | - | - |  | + | + |
| ribitol | C00474 |  | - | - |  | - | - |
| D-sorbitol | C00794 |  | - | - |  | - | - |
| trehalose | C01083 |  | + | + |  | + | + |

**Table S4**. (Continued)

| Carbon sources | KEGG |  | *Xanthomonas campestris* | |  | *Ralsonia solanacearum* | |
| --- | --- | --- | --- | --- | --- | --- | --- |
|  | entry ID |  | experimental | predicted |  | experimental | predicted |
| L-glutamate | C00025 |  | + | + |  | - | + |
| glucose | C00031 |  | + | + |  | + | + |
| glycine | C00037 |  | + | + |  | - | + |
| L-lysine | C00047 |  | + | + |  | - | - |
| L-aspartate | C00049 |  | + | + |  | + | + |
| L-arginine | C00062 |  | + | + |  | - | + |
| L-glutamine | C00064 |  | + | + |  | + | + |
| L-serine | C00065 |  | + | + |  | - | + |
| L-methionine | C00073 |  | + | + |  | + | + |
| L-tryptophan | C00078 |  | + | + |  | - | - |
| L-phenylalanine | C00079 |  | + | + |  | + | + |
| L-tyrosine | C00082 |  | + | + |  | + | + |
| sucrose | C00089 |  | + | + |  | + | + |
| D-fructose | C00095 |  | + | + |  | + | + |
| L-leucine | C00123 |  | - | - |  | - | - |
| L-histidine | C00135 |  | + | + |  | - | + |
| myo-inositol | C00137 |  | + | + |  | - | - |
| L-proline | C00148 |  | + | + |  | + | + |
| L-valine | C00183 |  | + | + |  | + | + |
| cellobiose | C00185 |  | + | + |  | - | - |
| L-threonine | C00188 |  | - | + |  | + | + |
| L-sorbose | C00247 |  | + | + |  | - | - |
| D-mannitol | C00392 |  | + | + |  | + | + |
| L-isoleucine | C00407 |  | - | + |  | - | - |
| pectate | C00470 |  | - | - |  | - | - |
| ribitol | C00474 |  | - | - |  | - | - |
| D-sorbitol | C00794 |  | - | - |  | - | - |
| trehalose | C01083 |  | - | + |  | - | - |

*Metabolizable carbon sources were predicted using KEGG PathComp.
