# Supplementary material for: New Detection Systems of Bacteria Using Highly Selective Media Designed by SMART: Selective Medium-Design Algorithm Restricted by Two Constraints
Source: PLoS One. 2011 Jan 27;6(1):e16512. doi: 10.1371/journal.pone.0016512 (PMC3029383; doi:10.1371/journal.pone.0016512)
Supplement: Table S5 — Antimicrobial resistance predicted by the NCBI database compared to experimental data. (DOC) [file pone.0016512.s008.doc]

**Table S5**. Antimicrobial resistance predicted by the NCBI database compared to experimental data.

|  |  | *Acidovorax avenae* | | |  | *Pectobacterium carotovorum* | | |
| --- | --- | --- | --- | --- | --- | --- | --- | --- |
| Antimicorbial | Reported Resistance | COG* | Predicted | Experimental |  | COG | Predicted | Experimental |
| Name | Gene Name |  | Resistance** | Data*** |  |  | Resistance | Data |
| ampicillin | beta-lactamase | COG2367V | R | R |  | - | S | S |
|  |  |  |  |  |  |  |  |  |
| cephalosporine | beta-lactamase | COG2367V | R | S |  | - | S | S |
|  |  |  |  |  |  |  |  |  |
| cetrimonium | quaternary ammonium compound | COG2076P | R | R |  | COG2076P | R | R |
|  | resistance protein |  |  |  |  |  |  |  |
|  |  |  |  |  |  |  |  |  |
| chloramphenicol | chloramphenicol | - | S | S |  | - | S | S |
|  | acetyltransferase |  |  |  |  |  |  |  |
|  | multidrug efflux pump *acrB* | - |  |  |  | - |  |  |
|  |  |  |  |  |  |  |  |  |
| gentamicin | aminoglycoside | - | S | S |  | - | S | S |
|  | phosphotransferase aac3 |  |  |  |  |  |  |  |
|  | aminoglycoside | - |  |  |  | - |  |  |
|  | adenyltransferase aadB |  |  |  |  |  |  |  |
|  |  |  |  |  |  |  |  |  |
| neomycin | aminoglycoside | - | S | S |  | - | S | S |
|  | phosphotransferase aac6 |  |  |  |  |  |  |  |
|  |  |  |  |  |  |  |  |  |
| penicillin | beta-lactamase | COG2367V | R | S |  | - | S | S |
|  |  |  |  |  |  |  |  |  |
| polymyxin | polymixin resistance | COG3173R | R | R |  | - | S | S |
|  | glycosyltransferase |  |  |  |  |  |  |  |
|  |  |  |  |  |  |  |  |  |
| streptomycin | streptomycin | - | S | S |  | - | S | S |
|  | phosphotransferase strA |  |  |  |  |  |  |  |
|  | streptomycin | - |  |  |  | - |  |  |
|  | phosphotransferase strB |  |  |  |  |  |  |  |
|  |  |  |  |  |  |  |  |  |
| trimethoprim | dihydrofolate reductase type I | - | S | S |  | - | S | R |
|  | dihydrofolate reductase type X | - |  |  |  | - |  |  |
|  |  |  |  |  |  |  |  |  |
| gramicidin | hydantoin racemase | - | S | S |  | COG4126E | R | R |

**Table S5**. (Continued)

|  |  | *Xanthomoas campestris* | | |  | *Ralstonia solanacearum* | | |
| --- | --- | --- | --- | --- | --- | --- | --- | --- |
| Antimicorbial | Reported Resistance | COG | Predicted | Experimental |  | COG | Predicted | Experimental |
| Name | Gene Name |  | Resistance | Data |  |  | Resistance | Data |
| ampicillin | beta-lactamase | COG2367V | R | S |  | - | S | S |
|  |  |  |  |  |  |  |  |  |
| cephalosporine | beta-lactamase | COG2367V | R | R |  | - | S | S |
|  |  |  |  |  |  |  |  |  |
| cetrimonium | quaternary ammonium compound | COG2076P | R | S |  | COG2076P | R | S |
|  | resistance protein |  |  |  |  |  |  |  |
|  |  |  |  |  |  |  |  |  |
| chloramphenicol | chloramphenicol | - | S | S |  | COG0110R | R | R |
|  | acetyltransferase |  |  |  |  |  |  |  |
|  | multidrug efflux pump *acrB* | - |  |  |  | COG0477GEPR |  |  |
|  |  |  |  |  |  |  |  |  |
| gentamicin | aminoglycoside | - | S | S |  | - | S | S |
|  | phosphotransferase aac3 |  |  |  |  |  |  |  |
|  | aminoglycoside | - |  |  |  | - |  |  |
|  | adenyltransferase aadB |  |  |  |  |  |  |  |
|  |  |  |  |  |  |  |  |  |
| neomycin | aminoglycoside | - | S | S |  | - | S | S |
|  | phosphotransferase aac6 |  |  |  |  |  |  |  |
|  |  |  |  |  |  |  |  |  |
| penicillin | beta-lactamase | COG2367V | R | R |  | - | S | S |
|  |  |  |  |  |  |  |  |  |
| polymyxin | polymixin resistance | - | S | S |  | COG3173R | R | R |
|  | glycosyltransferase |  |  |  |  |  |  |  |
|  |  |  |  |  |  |  |  |  |
| streptomycin | streptomycin | - | S | S |  | - | S | S |
|  | phosphotransferase strA |  |  |  |  |  |  |  |
|  | streptomycin | - |  |  |  | - |  |  |
|  | phosphotransferase strB |  |  |  |  |  |  |  |
|  |  |  |  |  |  |  |  |  |
| trimethoprim | dihydrofolate reductase type I | - | S | R |  | - | S | S |
|  | dihydrofolate reductase type X | - |  |  |  | - |  |  |
|  |  |  |  |  |  |  |  |  |
| gramicidin | hydantoin racemase | - | S | S |  | - | S | R |

*COG stands for clusters of orthologous groups of proteins (http://www.ncbi.nlm.nih.gov/COG/).

**R and S indicate resistant and susceptible, respectively.

***The concentration of antimicrobials added to the medium was 10 ppm.
