# Supplementary material for: New Detection Systems of Bacteria Using Highly Selective Media Designed by SMART: Selective Medium-Design Algorithm Restricted by Two Constraints
Source: PLoS One. 2011 Jan 27;6(1):e16512. doi: 10.1371/journal.pone.0016512 (PMC3029383; doi:10.1371/journal.pone.0016512)
Supplement: Table S6 — Determination of the appropriate agar and moisture concentration for colony formation on paper-based medium. (DOC) [file pone.0016512.s009.doc]

**Table S6**. Determination of the appropriate agar and moisture concentration for colony formation on paper-based medium.

| Addition of distilled water | Agar strength (g/L) | | | |
| --- | --- | --- | --- | --- |
| per filter paper |
| for keeping its moisture | 5.0 | 10.0 | 15.0 | 20.0 |
| 0 ul | 1/8* | 0/8 | 1/8 | 1/8 |
| 100 ul | 1/8 | 6/8 | 8/8 | 5/8 |
| 200 ul | 0/8 | 0/8 | 0/8 | 0/8 |
| 400 ul | 0/8 | 0/8 | 0/8 | 0/8 |

*Number of filters on which colonies formed / total number of filters tested.
